# Supplementary figures and images for: Risk factors for gastric perforation after cytoreductive surgery in patients with peritoneal carcinomatosis: Splenectomy and increased body mass index
Source: PLoS One. 2021 Mar 4;16(3):e0248205. doi: 10.1371/journal.pone.0248205 (PMC7932550; doi:10.1371/journal.pone.0248205)

**S1 Fig.** **Preservation of the gastroepiploic arcade during an omentectomy.**

**
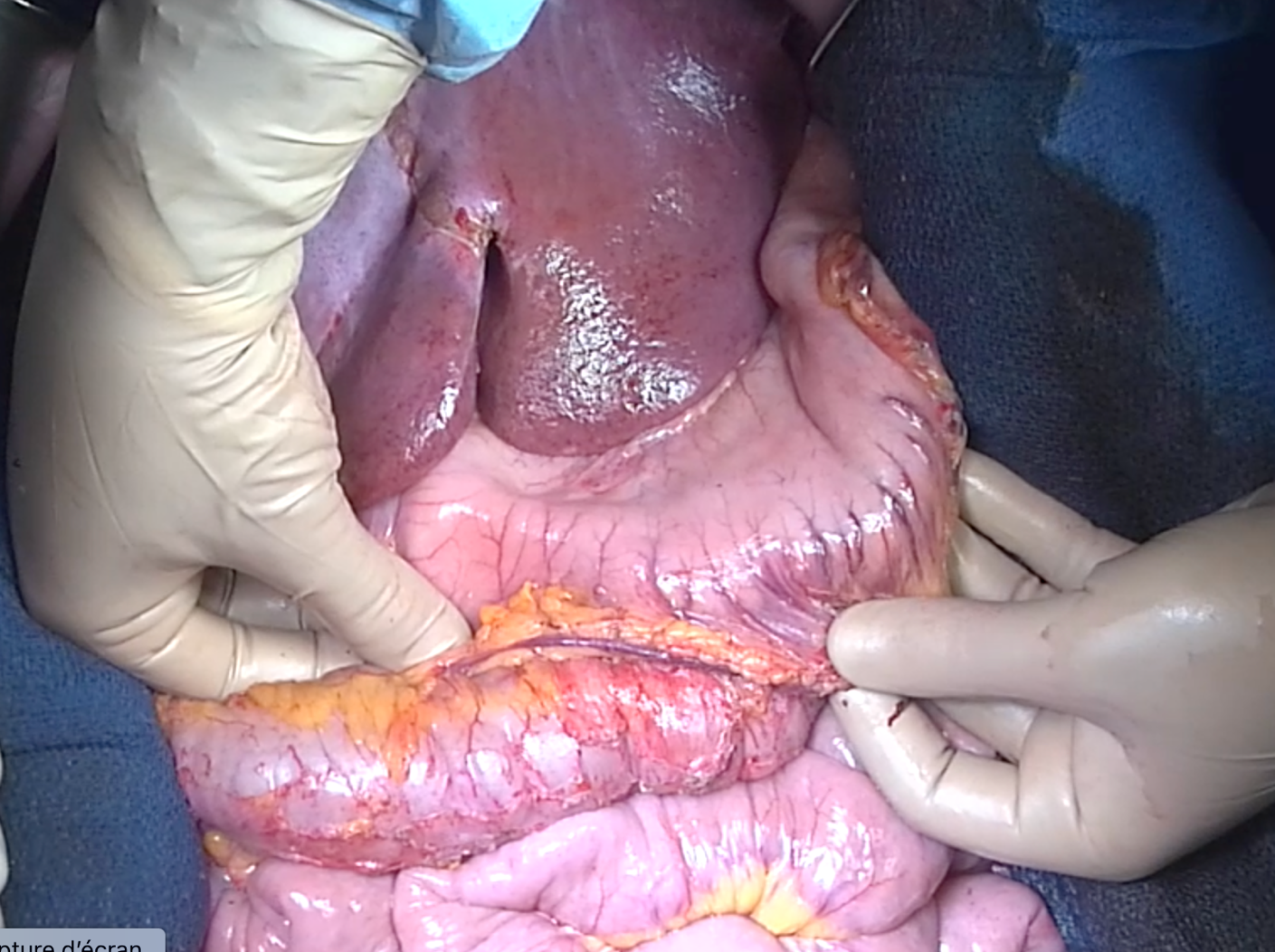
**

Supplement: S1 Fig — (DOCX) [file pone.0248205.s001.docx]

**S2 Fig.** **Prophylactic suture of the greater curvature of the stomach.**


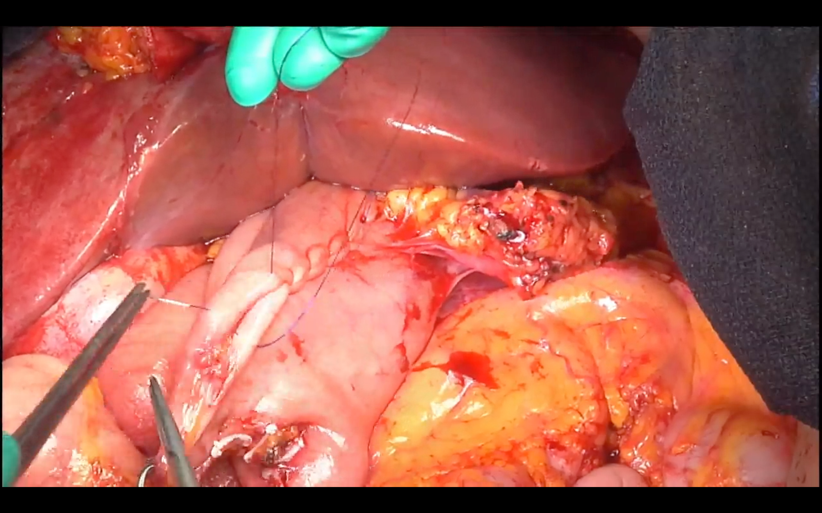

Supplement: S2 Fig — (DOCX) [file pone.0248205.s002.docx]
