# Supplementary material for: Risk factors for gastric perforation after cytoreductive surgery in patients with peritoneal carcinomatosis: Splenectomy and increased body mass index
Source: PLoS One. 2021 Mar 4;16(3):e0248205. doi: 10.1371/journal.pone.0248205 (PMC7932550; doi:10.1371/journal.pone.0248205)
Supplement: S3 Fig — (DOCX) [file pone.0248205.s003.docx]

**S3 Fig.** **Postoperative gastric perforation located in the upper portion of the greater curvature.**

**
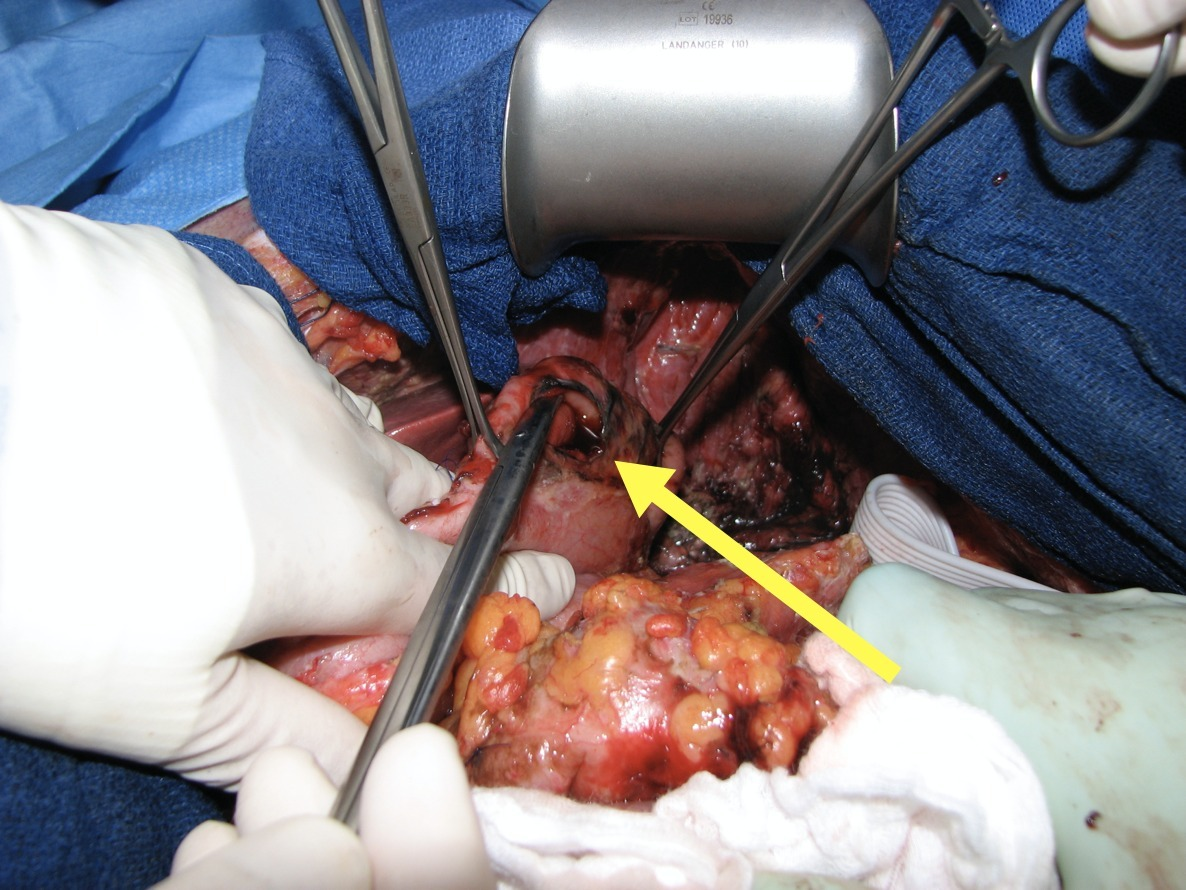
**
