# Supplementary material for: Risk factors for gastric perforation after cytoreductive surgery in patients with peritoneal carcinomatosis: Splenectomy and increased body mass index
Source: PLoS One. 2021 Mar 4;16(3):e0248205. doi: 10.1371/journal.pone.0248205 (PMC7932550; doi:10.1371/journal.pone.0248205)

**S4 Fig.** **Gastric opacified computed tomography.** Gastric wall perforation and extraluminal free air marked with yellow arrows.


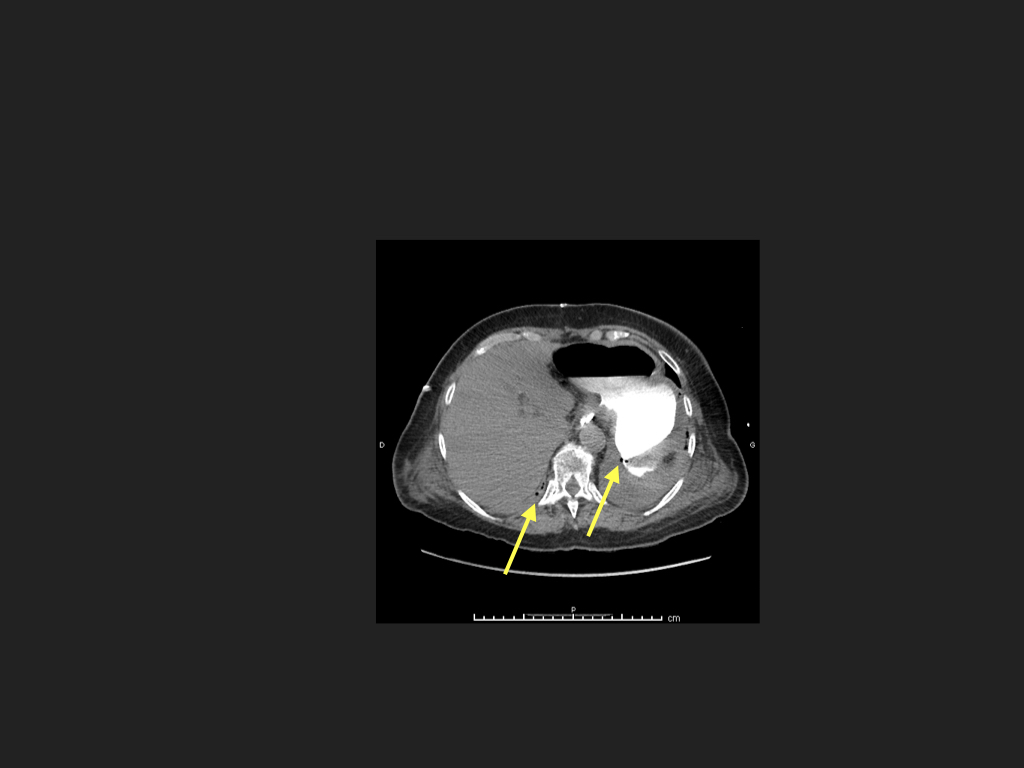

Supplement: S4 Fig — Gastric wall perforation and extraluminal free air are marked with yellow arrows. (DOCX) [file pone.0248205.s004.docx]
